# Non-chemical control of fungal pathogens in crops: a one-health perspective on strategies, mechanisms, and future directions

**DOI:** 10.3389/fpls.2025.1746521

**Published:** 2026-01-12

**Authors:** Charith Raj Adkar-Purushothama, Ashish Chettimada, Thokur Sreepathy Murali, Annamalai Muthusamy, Kamal Bouarab, Jean-Pierre Perreault

**Affiliations:** 1 Manipal School of Life Sciences, Manipal Academy of Higher Education, Manipal, Karnataka, India; 2 RNA Group/Groupe ARN, Département de Biochimie et de Génomique Fonctionnelle, Faculté de Médecine et des Sciences de la Santé, Pavillon de Recherche Appliquée au Cancer, Université de Sherbrooke, Sherbrooke, QC, Canada; 3 Centre SÈVE, Département de Biologie, Faculté des Sciences, Université de Sherbrooke, Sherbrooke, QC, Canada; 4 Department of Public Health Genomics, Manipal School of Life Sciences, Manipal Academy of Higher Education, Manipal, Karnataka, India; 5 Department of Plant Sciences, Manipal School of Life Sciences, Manipal Academy of Higher Education, Manipal, Karnataka, India

**Keywords:** biological control, fungal pathogens, induced resistance, microbiome engineering, pest management, RNA interference

## Abstract

Fungal pathogens threaten global crop production, food security, and environmental and human health. Though the reliance on chemical fungicides has provided effective control, but raises concerns over environmental contamination, toxic residues, and the rapid emergence of fungicide-resistant strains. These challenges, along with regulatory pressures, highlight the need for safer, more sustainable disease-management strategies. This review incorporates advances in non-chemical approaches for controlling fungal plant diseases, including cultural practices, biological control agents, natural plant metabolites, RNA-based technologies, nanotechnology, and microbiome engineering. We evaluate each strategy’s mechanisms, strengths, limitations, and remaining knowledge gaps. An integrated pest management framework is proposed to combine complementary methods, reduce dependence on chemical inputs, enhance crop resilience, and support human and ecosystem health.

## 1 Introduction

The One Health concept emphasizes the interdependence of human, animal, plant, and environmental health and is grounded in key principles such as prevention, systems thinking, transdisciplinary collaboration, and sustainable ecosystem stewardship (Lerner and Berg, 2017; Destoumieux-Garzón et al., 2018). Applied to plant fungal disease management, One Health encourages approaches that reduce chemical inputs, preserve ecological functions, and support safer food production systems. Strategies such as genetic resistance, biological control, induced immunity, cultural practices, and plant-derived antifungal compounds align with these principles by lowering pesticide use, protecting beneficial microbiota, reducing environmental contamination, and mitigating risks to human and animal health (Nicolopoulou-Stamati et al., 2016; Köhl et al., 2019). Integrating these methods within a One Health framework promotes resilient crops, biodiversity conservation, and sustainable agroecosystems, ultimately supporting the broader goal of maintaining health across interconnected biological and environmental domains.

Fungal pathogens pose a perennial and pervasive threat to crop production worldwide. Estimates suggest that diseases caused by fungi and oomycetes account for up to 70–80% of all crop disease losses, with direct economic losses reaching hundreds of billions of USdollars annually (Peng et al., 2021). The conventional toolbox for fungal disease management has centred on two pillars: deployment of host resistance (i.e., genetic cultivars) and the application of synthetic fungicides. The former includes breeding of genetic cultivars as well as the genetic modification of existing ones, although only a few GM crops with demonstrated field-level disease resistance exist most notably transgenic Cavendish banana expressing *RGA2*, which confers stable resistance to Fusarium wilt TR4 (Dale et al., 2017), and the late-blight-resistant Innate^®^ Gen2 potato carrying the *Rpi-vnt1* gene, which targets *Phytophthora infestans* (Health Canada, 2016, *Simplot Innate® Potato Event Gen2 Z6: Novel Food Information.* Government of Canada. Available online at: https://www.canada.ca/en/health-canada/services/food-nutrition/genetically-modified-foods-other-novel-foods/approved-products/simplot-innate-potato-event-gen2-z6.html [Accessed on December 10, 2025]). The latter consists of *de novo* synthesis of carbon-based compounds, which are not previously found in nature, and that are expressly manufactured to control/eliminate pest or pathogen populations. Yet, these control agents may drastically affect organisms other than being targeted, and may yield long-lasting residues, which exert adverse environmental effects beyond their intended use (Mir et al., 2023; Burandt et al., 2024). While both processes remain important, numerous obstacles to their application have emerged: (i) many field pathogens have developed resistance to key fungicide classes; (ii) chemical use is increasingly constrained by regulatory/consumer demand and environmental concerns; and (iii) durable genetic resistance is often lacking or can be rapidly overcome by pathogen evolution (Lucas et al., 2015; Ons et al., 2020; Singh et al., 2023). As a result, there is an urgent need to develop and deploy non-chemical approaches to fungal disease control that are environmentally sustainable, integrated with agronomic practices, and compatible with low-input or organic systems.

This review aims to summarize the status of non-chemical approaches to controlling fungal pathogens in crops, analyse the mechanisms by which they act, assess their strengths and limitations, and outline future research directions. We organize the review into major categories of approach: (1) cultural and agronomic practices; (2) host resistance and induced resistance; (3) biological control and microbial‐based strategies; (4) botanical and natural product fungicides; (5) advanced technologies (nanotechnology, RNA interference, and microbiome engineering); and (6) integration into pest management frameworks. In doing so, we highlight both established practices and emerging frontiers. In addition to highlighting the advantages of these strategies, we also examine the challenges and potential risks that advanced technologies may pose to environmental health.

## 2 Impact of chemical fungicides on the environment and health

Before examining alternative methods and reasons for their necessity, it is important to understand the key factors that are driving the shift toward non-chemical approaches. While chemical fungicides have been essential in crop protection, several major challenges have arisen due to their extensive and often indiscriminate use over the years (**Table 1**). The repeated use of fungicides with specific modes of action has placed tremendous selective pressures on fungal populations, leading to widespread development of resistance and reduced efficacy of many chemical controls. This is particularly evident in soil-borne vascular pathogens, such as *Fusarium oxysporum* Schlecht. emend. Snyder & Hansen (*Nectriaceae*) and *Verticillium dahliae* Kleb*. (Plectosphaerellaceae).* For these particular pathogens, no effective chemical treatments are currently available in many crops due to resistance or inherent limitations of fungicides (Song et al., 2020; Hudson et al., 2021; Li et al., 2023; Naqvi et al., 2025). In addition to resistance, fungicides may cause unintended harm to non-target organisms, including beneficial soil and plant-associated microbiota. For example, fungicide applications have been shown to reduce non-pathogenic phyllosphere yeast populations and alter overall microbial diversity, thereby potentially disrupting ecological balance (Noel et al., 2022). These ecological effects raise growing concerns among regulators and the public. Regulatory initiatives, such as the European Union’s Green Deal, aim to cut pesticide use by 50% by 2030, thereby reflecting both public health concerns and environmental priorities (https://food.ec.europa.eu/plants/pesticides/sustainable-use-pesticides_en; https://www.pan-europe.info; accessed: 15 October 2025). Certain pathogens, especially soil-borne and vascular fungi, can likewise pose unique challenges due to their persistence (e.g., through chlamydospores or microsclerotia), protected niches, and poor chemical accessibility, which limits fungicide translocation and effectiveness (Singh et al., 2023). These limitations highlight the urgent need for reliable and safer alternative disease management strategies. Consistent with the principles of sustainable agriculture, non-chemical approaches support system resilience by minimizing external inputs, while fostering beneficial microbial communities, enhancing innate plant defences, and promoting long-term ecological balance (McLaughlin et al., 2023).

**Table 1:** Effects of chemical fungicides on the environment and off-targets.

Fungicide class	Impact on the environment/off-targets	Effects observed	Key features / mechanism	Representative reference
General (various classes)	Soil microbial and nutrient cycling effects	Reduced microbial biomass, altered carbon/nitrogen cycling	Broad-spectrum fungicides suppress soil fungi, including beneficial mycorrhizae and decomposers, affecting nutrient dynamics	(Ullah and Dijkstra, 2019)
Azoles, strobilurins	Non-target organism toxicity (aquatic invertebrates)	Toxicity to aquatic invertebrates, such as *Asellus aquaticus*; impaired feeding and growth	Run-off leads to contamination of freshwater; fungicides affect aquatic fauna behaviourally and physiologically	(Mohan et al., 2024)
Broad-spectrum (e.g. Demethylation, Quinone outside Inhibitor fungicides)	Biodiversity / ecosystem effects	Reduced plant species diversity, disrupted fungal community balance	By suppressing both pathogenic and mutualistic fungi, fungicides change community structure in wild systems	(Wesche et al., 2025)
Copper-based (inorganic)	Persistence and environmental contamination	Accumulation of heavy metals in soil, long-term ecotoxicity	Copper-based fungicides persist in soil/sediment, causing toxicity to microbes, plants, and invertebrates	(Burandt et al., 2024)
Dithiocarbamates, benzimidazoles	Human and animal health effects	Potential endocrine disruption, carcinogenicity, reproductive toxicity	Occupational exposure and residues can affect human health via chronic low-dose exposure	(Campanale et al., 2023)
Azoles, multi-site inhibitors	Resistance development	Selection for fungicide-resistant pathogen strains	Continuous use drives resistance in pathogens (e.g. *Zymoseptoria*, *Aspergillus*), requiring stronger or more frequent applications	(Szczygieł et al., 2024)

The environmental contamination, microbial disruption, and human health risks associated with chemical fungicides, as detailed above, highlight the broader One Health implications of continued chemical dependence. These interconnected challenges spanning plant productivity, ecosystem integrity, and public health demonstrate that sustainable fungal disease management must extend beyond crop protection alone. Consequently, the non-chemical strategies that support a One Health framework by reducing chemical inputs, fostering ecological resilience, and promoting safer, healthier agricultural systems for both humans and the environment.

## 3 Available non-chemical strategies for fungal pathogen management

### 3.1 Ecologically based cultural practices for fungal disease management within a one health framework

#### 3.1.1 Cultural and agronomic practices for fungal pathogen management

Cultural and agronomic practices are foundational to integrate fungal disease management, which aims to reduce inoculum levels and create environmental conditions that are unfavourable to pathogen development. These include crop rotation with non-host species, fallow periods, sanitation, and residue management, which have been shown to suppress soil-borne pathogens, such as *F. oxysporum* and *V. dahliae* (Scott et al., 2014; Kowalska, 2021). Soil management techniques, such as improving drainage, reducing compaction, and adjusting pH, can limit disease-conducive conditions, while tillage practices can influence microbial communities, with no-till systems supporting higher microbial resilience (Noel et al., 2022). Optimizing plant spacing, canopy structure, and airflow reduces humidity and leaf wetness, suppressing foliar diseases (Douma and Noordhoek, 2025). Using disease-free seed material, enforcing quarantine, and applying non-chemical treatments can help prevent the introduction of pathogens (Mancini and Romanazzi, 2014). Thermal soil treatments, such as solarization and steam, combined with organic amendments, enhance suppressive soil microbial communities (Rippa et al., 2025). The use of compost and organic soil amendments can also promote microbial communities that are antagonistic to pathogens. These practices contribute to the development of disease-suppressive soils, where beneficial microorganisms inhibit pathogen activity and enhance rhizosphere resilience (Tziros et al., 2024; Rippa et al., 2025). Finally, practices such as intercropping, crop diversification, and cover cropping can reduce host continuity and promote beneficial microbes, leading to improved disease resistance and system resilience (Yu et al., 2022; Shrestha et al., 2025).

From a One Health perspective, these cultural and agronomic practices extend benefits beyond plant health by influencing ecosystem processes, human exposure, and overall agroecosystem resilience. By enhancing soil microbial diversity and nutrient cycling, promoting disease-suppressive soils, and reducing dependence on chemical fungicides, they minimize environmental contamination and occupational exposure risks for farm workers (Tziros et al., 2024; Douma and Noordhoek, 2025; Rippa et al., 2025). The use of disease-free planting material and non-chemical interventions further contributes to safer food systems and reduced human and animal health risks (Mancini and Romanazzi, 2014). Integrating these approaches fosters resilient agroecosystems that align plant disease management with broader One Health objectives, linking plant, environmental, and human well-being.

#### 3.1.2 Crop rotation, fallow, sanitation and residue management

Many fungal pathogens persist in plant debris or soil between cropping seasons, making inoculum reduction a key strategy in disease management. Crop rotation with non-host or poor-host species, the implementation of fallow periods, deep ploughing, and the removal or incorporation of infected residues are proven methods for decreasing disease pressure. These approaches are particularly effective against soil-borne pathogens such as *F. oxysporum* and *V. dahliae* (Fradin and Thomma, 2006; Scott et al., 2014; Kowalska, 2021). For example, *V. dahliae*, the causal agent of Verticillium wilt, poses a significant threat to crops such as tomato, potato, and cotton. The pathogen produces long-lived microsclerotia capable of surviving in soil for several years, thereby maintaining inoculum levels between cropping cycles. Rotating susceptible crops with non-host species, such as cereals (e.g., wheat or barley), for several seasons has been shown to substantially reduce the soil inoculum potential. Similarly, deep ploughing can help decrease disease incidence by burying infested residues and microsclerotia deeper into the soil profile, where environmental conditions are less conducive to survival (Kowalska, 2021). In the case of *F. oxysporum* f. sp. *lycopersici*, the causal agent of Fusarium wilt in tomato, the removal and destruction of infected plant material after harvest or the implementation of a fallow period have been demonstrated to effectively reduce inoculum levels and subsequent disease severity (Chitwood-Brown et al., 2021). In addition, using clean propagation material and adhering to strict sanitation practices helps prevent the introduction and buildup of fungal inoculum.

From a One Health perspective, inoculum-reduction strategies extend benefits beyond crop protection by influencing ecosystem health, human exposure, and agroecosystem resilience. Crop rotation, fallow periods, residue management, and deep ploughing not only suppress soil-borne pathogens but also enhance soil microbial diversity and nutrient cycling, supporting disease-suppressive soils (Scott et al., 2014; Kowalska, 2021). By reducing the need for chemical fungicides, these practices lower environmental contamination and decrease occupational exposure risks for farm workers, as well as potential residues in food and water systems (Fradin and Thomma, 2006). The use of disease-free propagation material and strict sanitation prevents pathogen introduction, contributing to safer food production and reduced human and animal health risks (Mancini and Romanazzi, 2014; Chitwood-Brown et al., 2021). Collectively, these interventions create more resilient agroecosystems, linking crop-level disease management decisions to broader ecosystem processes and One Health outcomes. Integrating such practices highlights the cross-domain benefits of cultural and agronomic strategies, aligning sustainable disease control with plant, environmental, and public health objectives.

#### 3.1.3 Tillage, soil management and drainage

Soil structure, health, and moisture management play critical roles in fungal pathogen dynamics. Practices that improve drainage, prevent compaction, and enhance soil tilth can reduce pathogen proliferation. Adjusting soil pH and increasing organic matter content can also make the environment less conducive to fungal disease proliferation. Notably, a study comparing no-till and conventional systems found that no-till practices improved microbial resilience to fungicide applications, indicating the potential of certain tillage systems to support disease-suppressive microbial communities (Noel et al., 2022). From a One Health perspective, these soil management practices extend benefits beyond plant health by influencing ecosystem functions, human exposure, and agroecosystem resilience. Well-structured and biologically active soils support diverse microbial communities that enhance nutrient cycling, suppress pathogens, and reduce the need for chemical fungicides, thereby lowering environmental contamination and occupational exposure risks. By preventing the accumulation of pathogen inoculum and promoting ecological stability, these interventions contribute to broader agroecosystem resilience and link local disease management decisions to cross-domain One Health outcomes, benefiting plants, people, and the environment.

#### 3.1.4 Optimized planting, spacing and canopy management

Plant density and canopy structure markedly influence microclimatic conditions such as humidity and leaf wetness, which are critical for the development of many foliar fungal pathogens. Agronomic practices include proper plant spacing, pruning, trellising, and thinning that improve airflow and reduce humidity, thereby suppressing disease development without chemical input. These strategies are especially important in high-value fruit and vine crops where canopy microclimate plays a major role in pathogen dynamics (Douma and Noordhoek, 2025). From a One Health perspective, optimizing plant density and canopy structure provides benefits that extend beyond plant disease suppression. By reducing leaf wetness and humidity, these practices limit foliar pathogen development while minimizing the need for chemical fungicides, thereby lowering environmental contamination and potential human exposure. Improved airflow and canopy management also support beneficial microbial communities on leaf surfaces and in the rhizosphere, contributing to ecosystem health and resilience. Integrating these canopy-focused practices within a One Health framework demonstrates interconnected benefits for plant, environmental, and human health outcomes, while enhancing crop quality and yield stability.

#### 3.1.5 Use of disease-free seed/propagules and quarantine

Preventing the introduction of fungal pathogens through contaminated planting material is a critical preventive strategy. Seed- and propagule-borne fungi can cause systemic infections and rapid disease spread if not managed at the source. The use of disease-free material, non-chemical seed treatments, nursery sanitation, and quarantine enforcement is effective in minimizing this risk (Mancini and Romanazzi, 2014; Hamelin et al., 2021). This is particularly vital in crops relying upon vegetative propagation, where pathogens can persist asymptomatically. From a One Health perspective, using disease-free seed and propagules provides benefits that extend beyond plant protection. Preventing the introduction of fungal pathogens reduces the need for chemical interventions, thereby minimizing environmental contamination and limiting human and animal exposure. Nursery sanitation, quarantine enforcement, and non-chemical treatments support healthy microbial communities in soils and propagation systems, enhancing ecosystem resilience. By controlling pathogens at the source, these measures also contribute to safer food production and lower risks of pathogen spread across agricultural landscapes. Integrating clean planting material within a One Health framework highlights the interconnected benefits for plant, environmental, and human health outcomes.

#### 3.1.6 Host management through crop diversification, intercropping and cover crops

Diversifying cropping systems through intercropping, rotation, and cover cropping interrupts pathogen life cycles by reducing continuous host availability. These practices also enhance soil microbial diversity, which supports disease suppression through mechanisms like resource competition, antagonism and stimulation of plant immunity. For example, leguminous cover crops have been shown to improve soil fungal community composition by increasing beneficial fungi and reducing pathogen populations (Boudreau, 2013; Yu et al., 2022; Shrestha et al., 2025). Overall, these strategies build ecological stability and align with long-term goals of sustainable disease management.

Cultural practices offer several important strengths in plant disease management. They are generally low-cost, widely accessible to farmers, and form a core component of integrated pest management (IPM) systems (Pretty and Bharucha, 2015; Wang et al., 2021). Practices such as crop rotation, sanitation and optimal planting arrangements can substantially reduce disease risk and inoculum levels, making them a sustainable first line of defence. Yet, these approaches also have evident limitations. Their effectiveness is often partial and slower acting compared to chemical controls, and they typically require system-level changes and consistent implementation of good agronomic practices to be effective (Lichtfouse et al., 2009). In high disease pressure scenarios, cultural methods alone may not provide sufficient control and are best used in combination with other strategies. Nevertheless, they serve as an essential foundation for layering on additional non-chemical tools, contributing to more resilient and environmentally sound crop protection systems.

From a One Health perspective, crop diversification, intercropping, and cover cropping provide benefits that extend beyond direct disease suppression. By enhancing soil microbial diversity and promoting beneficial fungi, these practices improve ecosystem functions such as nutrient cycling and disease-suppressive soil formation (Boudreau, 2013; Yu et al., 2022; Shrestha et al., 2025). Reducing continuous host availability and pathogen pressure decreases reliance on chemical fungicides, lowering environmental contamination and minimizing human and animal exposure to harmful residues. These practices also contribute to agroecosystem resilience, supporting long-term productivity, soil health, and adaptive capacity under changing climatic conditions. When integrated with sanitation, optimal planting arrangements, and other cultural methods, host management strategies foster multi-layered disease control that aligns plant protection with broader health and sustainability outcomes. Framing these interventions within a One Health model explicitly links agricultural management choices to cross-domain benefits for plants, people, and the environment.

### 3.2 Host resistance and induced resistance

Harnessing the plant’s own defence mechanisms provides a sustainable strategy for controlling fungal pathogens within integrated, non-chemical frameworks. These approaches either leverage natural or engineered genetic resistance or activate induced resistance pathways to strengthen plant immunity prior to pathogen attack (Walters et al., 2013; Fenster and Eckert, 2021). By reducing reliance on chemical fungicides, they contribute to safer, more resilient agroecosystems that align plant protection with environmental and human health objectives.

#### 3.2.1 Genetic host resistance

Genetic host resistance remains a cornerstone of sustainable disease management, providing a first line of defence against fungal pathogens (Dangl et al., 2013). Both qualitative (major gene) and quantitative (polygenic) traits can substantially reduce disease burden, yet resistance is often overcome by rapidly evolving pathogens, particularly in monocultures or when deployed without complementary strategies. Integrating host resistance with cultural, biological, and agronomic interventions reduces selection pressure on pathogens and prolongs resistance durability, while simultaneously lowering the need for chemical fungicides. This integration contributes to a One Health framework by protecting soil and water quality, reducing human and animal exposure to harmful chemicals, and supporting resilient agroecosystems that maintain productivity and ecosystem services (Turrà et al., 2014; Köhl et al., 2019).

#### 3.2.2 Induced resistance

Induced resistance and defence priming offer additional layers of protection. This involves sensitizing the plant’s immune system through prior exposure to specific stimuli, such as microbial signals, plant extracts, or signalling molecules like salicylic acid, jasmonic acid, and their analogues. These signals prime the plant to mount a more rapid and stronger defence upon challenged by a pathogen. Mechanistically, induced resistance involves the activation of key defence pathways, including the accumulation of pathogenesis-related (PR) proteins, reactive oxygen species (ROS), phytoalexin production, cell wall reinforcement (e.g., callose deposition), and systemic responses, such as systemic acquired resistance (SAR) and induced systemic resistance (ISR) (van Verk et al., 2009; Pieterse et al., 2012). These mechanisms can markedly reduce disease severity even when there is no direct antagonism by the pathogen (Pieterse et al., 2014).

From a One Health perspective, induced resistance and defence priming not only enhance plant immunity but also influence broader ecosystem and human health outcomes. By reducing disease severity without relying on chemical fungicides, these strategies minimize environmental contamination and occupational exposure risks for farm workers, while lowering residues in food and water systems (Walters et al., 2013; Köhl et al., 2019). The activation of plant defence pathways can also affect soil and rhizosphere microbial communities, promoting beneficial microbes that contribute to nutrient cycling and disease-suppressive soils (Korenblum et al., 2022). Furthermore, resilient plants with primed defences may maintain productivity under pathogen pressure, supporting stable food supplies and ecosystem services. Integrating induced resistance with complementary cultural, biological, and genetic strategies thus exemplifies a cross-domain One Health approach, linking plant health, environmental integrity, and human well-being (van Verk et al., 2009; Pieterse et al., 2014).

#### 3.2.3 Elicitors and defence-activating compounds

**Figure 1:**
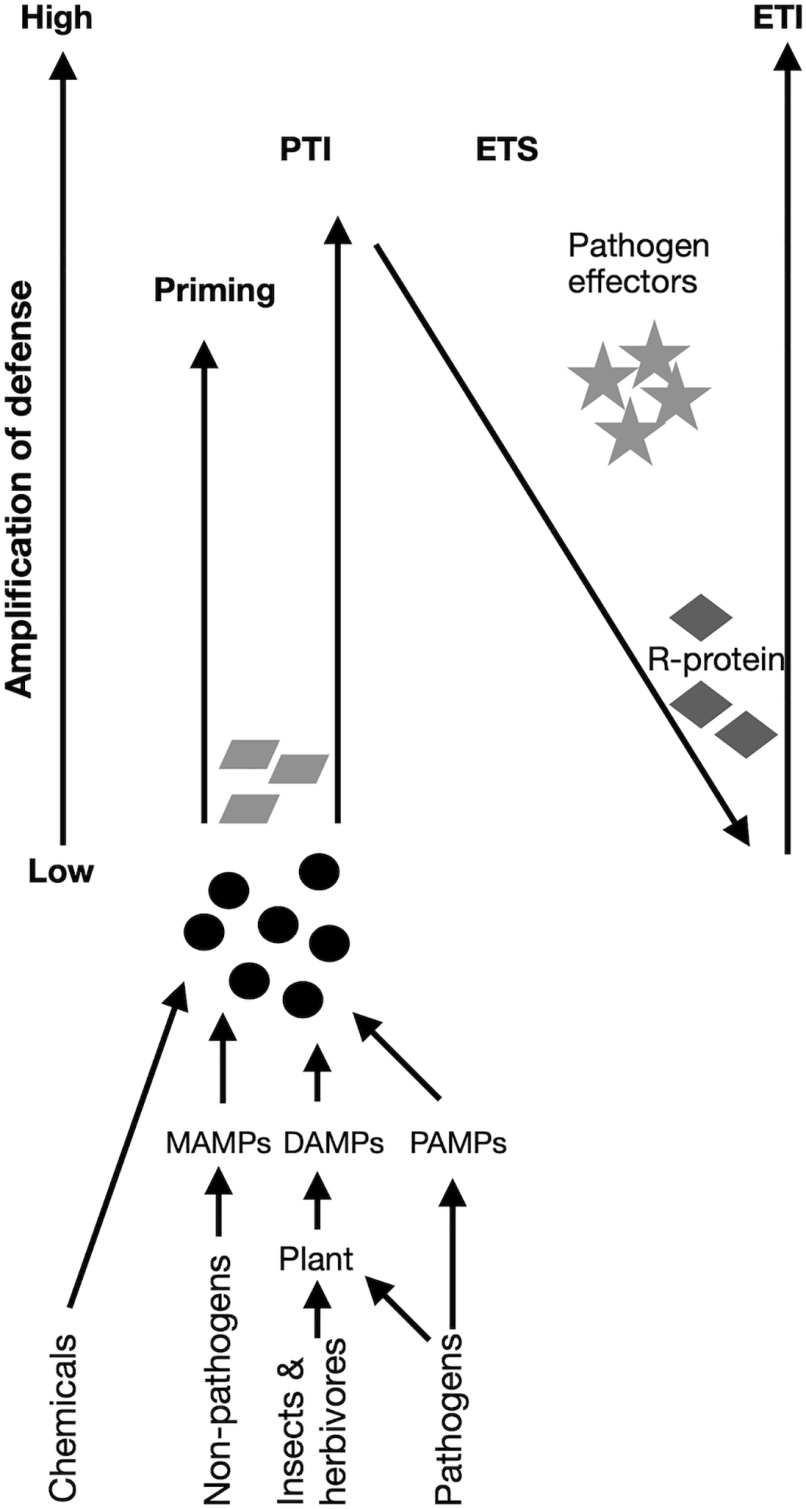
Compounds that trigger elicitors and defence-activating compounds in plants. Recognition leads to the onset of defence mechanisms that are referred to as pattern-triggered immunity (PTI). Adapted pathogens secrete effectors that disturb plant defence mechanisms, leading to effector-triggered susceptibility (ETS). Plant resistance (R) proteins recognize pathogen effectors and induce effector-triggered immunity (ETI). Treatment of plants with elicitor compounds (chemicals, microbe-associated molecular patterns (MAMPS) derived from non-pathogenic microbes, pathogen-associated molecular patterns (PAMPS) derived from pathogens, and damage-associated molecular patterns (DAMPS) that are produced by plants upon insect, herbivore, or pathogen attack via transmembrane pattern recognition receptors (PRRs). MAMPs, DAMPs, or PAMPs in the absence of an adapted pathogen leads to priming or PTI-based immunity that keeps plants to be in a heightened state of defence that provides some enhanced resistance toward otherwise virulent pathogens. Figure and its legend adapted from (Wiesel et al., 2014) under CC-BY 4.0.

In addition to genetic host resistance and inducing defence priming, elicitors and defence-activating compounds such as β-aminobutyric acid (BABA), chitosan, and various plant-derived molecules can be applied as foliar sprays or seed treatments to trigger plant defences artificially. These inputs bolster the plant’s immunity rather than targeting the pathogen itself and, therefore, are less likely to contribute to resistance development (Walters et al., 2013). The schematic representation presented in **Figure 1** depicts various compounds that trigger elicitors and defence-activating compounds in plants. These strategies come with both strengths and limitations. On the positive side, defence-based methods are less likely to drive pathogen resistance, are compatible with other interventions, and can be applied prophylactically. On the negative side, their efficacy can vary with crop species, environmental conditions, and the specific pathogen that is involved. In some cases, they may only offer partial suppression rather than full control, and the cost or regulatory approval of certain elicitors may limit their widespread use (Fenster and Eckert, 2021).

In practice, the most effective disease suppression often results from combining genetic resistance (both constitutive and inducible) with microbial biocontrol agents and cultural practices. This layered approach reduces dependence upon any single method and contributes to more resilient, sustainable crop protection systems. From a One Health perspective, elicitors and defence-activating compounds represent plant-centred interventions that enhance host immunity while minimizing direct ecological and human exposure to antimicrobial agents. Compounds such as β-aminobutyric acid and chitosan activate endogenous defence pathways, reducing disease pressure without exerting strong selective pressure on pathogens and thereby lowering risks of resistance development (Walters et al., 2013). Because these strategies do not rely on direct toxicity, they can reduce chemical inputs and associated environmental contamination, with potential benefits for non-target organisms and occupational exposure of farm workers. However, their context-dependent efficacy and partial disease suppression necessitate integration with genetic resistance, microbial biocontrol and cultural practices to ensure consistent field performance (Fenster and Eckert, 2021). When deployed within integrated systems, defence-activating compounds contribute to resilient crop protection frameworks that align plant immunity, ecosystem integrity and human health objectives under a One Health model. The next section of this review will explore microbial and plant product-based interventions, which constitute most non-chemical strategies.

### 3.3 Biological control and microbial-based strategies

Biological control (biocontrol) utilizes living organisms such as beneficial bacteria, fungi, and viruses to suppress plant pathogens and represents one of the most dynamic and rapidly evolving areas of non-chemical disease management. In the context of fungal pathogens, biocontrol includes antagonistic bacteria and fungi, endophytes, mycoviruses, microbial consortia, and microbiome manipulation, offering diverse mechanisms to reduce disease incidence and severity (Hacquard et al., 2017; Fontana et al., 2021; Boro et al., 2022). A broad range of antagonistic microorganisms has been explored as biocontrol agents (BCAs). Beneficial bacteria, particularly those in the genera *Bacillus*, *Pseudomonas (Pseudomonadaceae)*, and *Streptomyces (Streptomycetaceae)*, and ascomycete fungi such as *Trichoderma* (*Hypocreaceae*) and *Chaetomium (*Chaetomiaceae*)* species, have shown efficacy against various fungal pathogens. Their modes of action are multifaceted, including competition for nutrients and space, secretion of anti-fungal metabolites such as lipopeptides and antibiotics (e.g., iturins, fengycins and surfactins that are produced by *Bacillus* and *Pseudomonas*), hydrolytic enzymes like chitinases and glucanases that degrade fungal cell walls, induction of systemic resistance in plants, and mycoparasitism where fungi attack other fungi (Harman et al., 2004; Ongena and Jacques, 2008). As noted elsewhere, broader success of this “vaccination effect” has been accrued to several crops through the increasing availability of commercialized *Bacillus* preparations (e.g., Serenade and Sonata), which have captured one-third of the organic disease control market (Rhouma, 2025). In another example, the fungus *Chaetomium cupreum* L.M Ames produces both hydrolytic enzymes and anti-fungal compounds and has been commercialized as a biocontrol product (Zhang and Yang, 2007; Linkies et al., 2021). The plant endosphere and rhizosphere microbiomes host complex microbial communities that substantially influence plant health and disease resistance. Endophytic microbes can enhance host defence or antagonize pathogens directly. In addition to improving the host fitness, the secondary metabolites produced by the endophytes can also prevent pathogen entry (Bard et al., 2024). Manipulating these microbial communities by introducing beneficial strains or synthetic microbial consortia is a frontier area in biocontrol research (Medison et al., 2022). Recent advances highlight the potential of microbiome engineering to develop disease-suppressive soils, soils where pathogens are present, but disease incidence is low due to beneficial microbial interactions (Berg et al., 2020). Engineering microbial consortia to create stable, beneficial communities in the field offers promising new tools for sustainable disease.

Mycoviruses (i.e., viruses that infect fungal pathogens and reduce their virulence, hypovirulence), provide yet another biological avenue for disease management. Exploiting mycoviruses or fungal hyperparasites can decrease pathogen fitness and disease severity, although their practical application remains relatively nascent compared to other biocontrol strategies (García-Pedrajas et al., 2019; Khan et al., 2023; Galli et al., 2025). Recent mechanistic and molecular advances are expanding the biocontrol toolkit. These developments include genetically engineering BCAs to enhance their effectiveness, employing RNA interference (RNAi) technologies to silence essential pathogen genes, and targeting fungal efflux transporters to restore sensitivity to fungicides (Kristiawan et al., 2016; Pegg et al., 2020). These approaches blur the traditional boundaries between biological and molecular pest control.

Numerous case studies demonstrate biocontrol’s practical application. *The Solanaceae* includes important crop staples: peppers (*Capsicum* spp.), potato (*Solanum tuberosum* L.), tomato (*S. lycopersicum* L.), eggplant or aubergine (*S. melongena* L.), tomatillo or ground cherry (*Physalis philadelphica* Lamarck), and goji berry (*Lycium barbarum* L., *L. chinense* Miller). A recent review focusing upon crop species within this family highlighted the successful use of *Trichoderma* spp., *Bacillus* spp., *Pseudomonas fluorescens (Flügge) Migula (Pseudomonadaceae) Beauveria bassiana (*Bals.-Criv.*)* Vuill. (*Cordycipitaceae*), and *Gliocladium* spp. (Hypocreaceae), which are often found in consortia or integrated with other disease management tactics, underscoring the benefits of combining microbial agents with cultural and host resistance strategies (Jia et al., 2023; Madlhophe et al., 2025). The advantages of biocontrol include environmental friendliness, compatibility with organic and low-input systems, a lower risk of resistance development by pathogens, and additional benefits such as plant growth promotion and improved stress tolerance (Köhl et al., 2019). However, challenges remain in that biocontrol efficacy can be inconsistent under field conditions due to dependence on environmental factors like soil type, temperature, and moisture. Given the complexity of interactions among various factors, insufficient specificity in biocontrol applications, i.e., adverse effects on non-target species, may arise in undesirable consequences for both beneficial organisms and ecosystems (Villavicencio-Vásquez et al., 2025). Effective formulation, delivery methods, shelf life, and regulatory hurdles also constrain the adoption of these practices. Achieving consistent, large-scale field performance remains a critical bottleneck (Compant et al., 2019). Therefore, further research is required to understand the context dependency of microbial biocontrol agents performance (see **Table 2**), optimizing formulations and delivery systems, engineering stable synthetic microbial consortia for field application, assessing ecological risks or non-target effects, and integrating biocontrol with other management practices such as host resistance and cultural methods to maximize their synergistic effects (Miller et al., 2019; Berg et al., 2020).

**Table 2:** The major selected microbial biocontrol agents (BCAs) for fungal pathogens and their mechanisms.

BCA (organism)	Host crop / target fungal pathogen(s)	Key mechanisms of action	Comments / notes	Reference
*Trichoderma spp.* (fungus)	Numerous crops; soil-borne pathogens (e.g., *Fusarium*, *Rhizoctonia*, *Botrytis*)	Mycoparasitism, production of hydrolytic enzymes (chitinases/glucanases), competition for nutrients/space, induction of plant systemic resistance	Widely used, broad-spectrum; requires good formulation/soil conditions.	(Villavicencio-Vásquez et al., 2025)
*Bacillus* spp. (bacterium)	Various crops; fungal pathogens (soil- and foliar-borne)	Production of lipopeptides (iturin, surfactin, fengycin), volatile organic compounds (VOCs), induction of plant defences, competition	Good potential; field consistency and delivery remain challenges.	(Villavicencio-Vásquez et al., 2025)
*Paecilomyces spp.* (fungus)	Wilts, damping-off diseases in various hosts	Anti-fungal non‐volatile compounds, VOCs, hydrolytic enzymes, competition, induction of plant resistance	Emerging BCA; safety (toxins/mycotoxins) and commercialization require further study.	(Shi et al., 2025)
Yeasts (e.g., *Aureobasidium pullulans*, *Candida oleophila*)	Post-harvest, fruit/vegetable fungal spoilage; some foliar diseases	Competition for space/nutrients, VOCs, enzyme secretion, induction of host resistance	Good in post-harvest context; less data for field soil/foliar systems.	(Pandit et al., 2022)
Mycoviruses (viruses infecting plant-pathogenic fungi)	Target fungal pathogens via hypovirulence	Virus infection reduces pathogen virulence, slows pathogen growth/fitness	Highly promising but still experimental; ecological risks and delivery remain open issues.	(Galli et al., 2025)

From a One Health perspective, biological control and microbial-based disease management act as cross-domain interventions linking plant health with ecosystem function and human and animal exposure pathways. Beneficial bacteria, fungi, endophytes and engineered microbial consortia suppress fungal pathogens while reinforcing soil biodiversity and microbial processes that support nutrient cycling and disease-suppressive soils (Hacquard et al., 2017; Berg et al., 2020). Reduced reliance on chemical fungicides can lower environmental contamination and occupational exposure risks and decrease fungicide residues in food and water systems (Köhl et al., 2019). However, One Health-aligned deployment requires careful assessment of non-target and off-site effects, as introduced or engineered microbes may alter the native microbial communities with cascading ecological consequences (Compant et al., 2019; Villavicencio-Vásquez et al., 2025). Advances in microbiome engineering, mycovirus-based control and RNAi technologies offer routes to improve specificity and stability, enabling disease control strategies that enhance agroecosystem resilience while minimizing ecological disruption (García-Pedrajas et al., 2019; Berg et al., 2020).

### 3.4 Plant-derived compounds and natural-product fungicides

Another important category of non-chemical control strategies against fungal pathogens involves the use of plant-derived compounds, including botanical extracts, essential oils, and naturally occurring bioactive molecules. These compounds possess anti-fungal properties and can be applied in various forms such as foliar sprays, seed treatments, coatings, or soil amendments, thereby making them versatile tools in disease management programs. Plant-derived fungicides are increasingly examined not only for their efficacy against plant pathogens but also for their broader One Health implications.

Botanical extracts and essential oils are rich in secondary metabolites like terpenes, phenolics, flavonoids, and alkaloids that can suppress fungal growth by inhibiting spore germination, disrupting cell membranes, or interfering with fungal metabolism. The complexity of these natural mixtures is a key strength, as it reduces the likelihood of resistance development by targeting pathogens through multiple biochemical pathways. A recent review emphasized that botanical-based products impose a lower selective pressure on pathogens compared to synthetic fungicides with a single mode of action. For instance, essential oils that are extracted from *Mentha x piperita* L. (peppermint; *Lamiaceae*), *Foeniculum vulgare* Miller (fennel; Apiaceae), *Coriandrum sativum* L. (coriander, Apiaceae), and *Allium ascalonicum* L. (shallot; Amaryllidaceae) have shown promising anti-fungal activity against various plant pathogens (Kowalska, 2021).

Commercialization of botanical fungicides has begun, although these products are still in early phases of adoption compared to their chemical counterparts. A recent review notes that despite their potential, challenges such as a lack of standardization, limited stability, high variability between batches, and regulatory barriers hinder the wider use of extracts and essential oils in this control context (Bhandari et al., 2021). Standardizing extraction processes, ensuring consistency in active compound concentrations, and improving shelf life are necessary for their broader market acceptance.

The mechanisms of action of these plant-derived products are diverse and include disruption of fungal membrane integrity, inhibition of spore germination, suppression of enzymatic and metabolic activity in fungi, and stimulation of the plant’s own defence responses (e.g., through defence gene activation or hormonal signalling). These multi-target effects enhance their utility in integrated pest management (IPM) systems and make them less prone to resistance problems compared to synthetic fungicides that target specific metabolic activity (Ons et al., 2020; Reddy and Chowdary, 2021; Silva-Beltrán et al., 2023). Yet, the potency of plant-derived compounds can vary depending on plant species, extraction method, and formulation. Furthermore, these products often provide only partial disease control, and their efficacy under field conditions remains variable. Problems such as cost, storage stability, and regulatory approval also limit their scalability and commercial adoption. Moreover, batch-to-batch variability and environmental sensitivity can complicate formulation and application (Deresa and Diriba, 2023). These issues can be addressed by integrating plant-derived products with other non-chemical strategies (e.g., microbial biocontrol or induced resistance), which offer potential for synergistic effects and more robust disease management frameworks (Deresa and Diriba, 2023).

Within a One Health framework, the use of plant-derived compounds as fungicides represents a disease management choice with cascading benefits across plant, environmental, and human health domains. Botanical extracts and essential oils exhibit multi-target antifungal activity while generally degrading more rapidly in soil and water than synthetic fungicides, thereby reducing ecological persistence and minimizing disruption to beneficial soil microbial communities involved in nutrient cycling and plant resilience (Ons et al., 2020; Kowalska, 2021). Lower environmental persistence and toxicity also decrease off-target exposure risks for non-target organisms, including pollinators and aquatic species, contributing to ecosystem stability (Silva-Beltrán et al., 2023). From a human health perspective, reduced reliance on synthetic fungicides may lower occupational exposure among farm workers and decrease pesticide residues in food, with potential implications for long-term health outcomes (Reddy and Chowdary, 2021). Although challenges related to variability, formulation stability, and regulatory approval remain, integrating plant-derived fungicides with complementary non-chemical strategies can enhance disease control while aligning crop protection practices with One Health objectives that emphasize sustainability, exposure reduction, and system-wide resilience (Bhandari et al., 2021; Deresa and Diriba, 2023).

### 3.5 Advanced technologies: nanotechnology, RNAi and microbiome engineering

Beyond traditional non-chemical methods, emerging advances in biotechnology, nanoscience, and microbiome engineering are reshaping the landscape of sustainable fungal disease management. These technologies offer new avenues for precision control, lower chemical dependency, and targeted delivery of protective agents. These precision and targeted strategies enhance crop protection while promoting healthier soils, resilient ecosystems, and safer food production. By integrating advanced technologies with sustainable practices, they help safeguard both public health and environmental integrity.

#### 3.5.1 Nanotechnology

Nanotechnology has shown significant potential using biologically synthesized nanoparticles (NPs), nano-carriers, and nano-formulations of anti-fungal compounds. These systems enhance the targeted delivery, stability, and controlled release of active ingredients, allowing for lower application rates and reduced environmental impacts. A recent review highlights the efficacy of metallic nanoparticles (e.g., silver and copper), graphene-based nanomaterials, and nanocomposites against major fungal pathogens such as *F. oxysporum* and *Botrytis cinerea* (Balusamy et al., 2023). These nanoparticles disrupt the fungal cell membranes, generate reactive oxygen species (ROS), and facilitate deeper penetration of active agents into the pathogen’s tissues. Nano-formulations are also being designed to encapsulate beneficial microbes or RNA molecules, enhancing both their efficacy and environmental persistence (Khundi et al., 2025). Though nanotechnology offers powerful tools for plant disease management, but its environmental risks must be carefully considered within a One Health framework. Metallic nanoparticles such as silver and copper can accumulate in soil and water, potentially disrupting beneficial microbial communities that underpin soil health and ecosystem stability (Maurer-Jones et al., 2013). Their ability to generate reactive oxygen species (ROS), while effective against pathogens, may also exert toxicity on non-target organisms, including soil invertebrates, aquatic species, and plant-associated microbiota (Khodakovskaya et al., 2009). Concerns have also been raised about the long-term persistence, bioaccumulation, and trophic transfer of nanoparticles in agroecosystems, which could indirectly affect animal and human health (Lead et al., 2018). Assessing nanoparticle fate and ecological effects is therefore critical to ensuring that nanotechnology supports, rather than undermines, One Health goals for safe, sustainable plant disease management.

#### 3.5.2 RNA interference in fungal plant pathogen control

RNA interference (RNAi) is an advanced molecular technology that has gained significant attention as a promising strategy for controlling fungal plant pathogens. This naturally occurring gene-silencing mechanism is activated by the presence of double-stranded RNA (dsRNA), which triggers the degradation of complementary messenger RNA (mRNA), thereby inhibiting the expression of specific target genes. First discovered in the free-living nematode *Caenorhabditis elegans* (Fire et al., 1998), RNAi has since been adapted in plant pathology to silence genes crucial for the growth, virulence, or survival of phytopathogenic fungi (Rosa et al., 2018).

**Figure 2:**
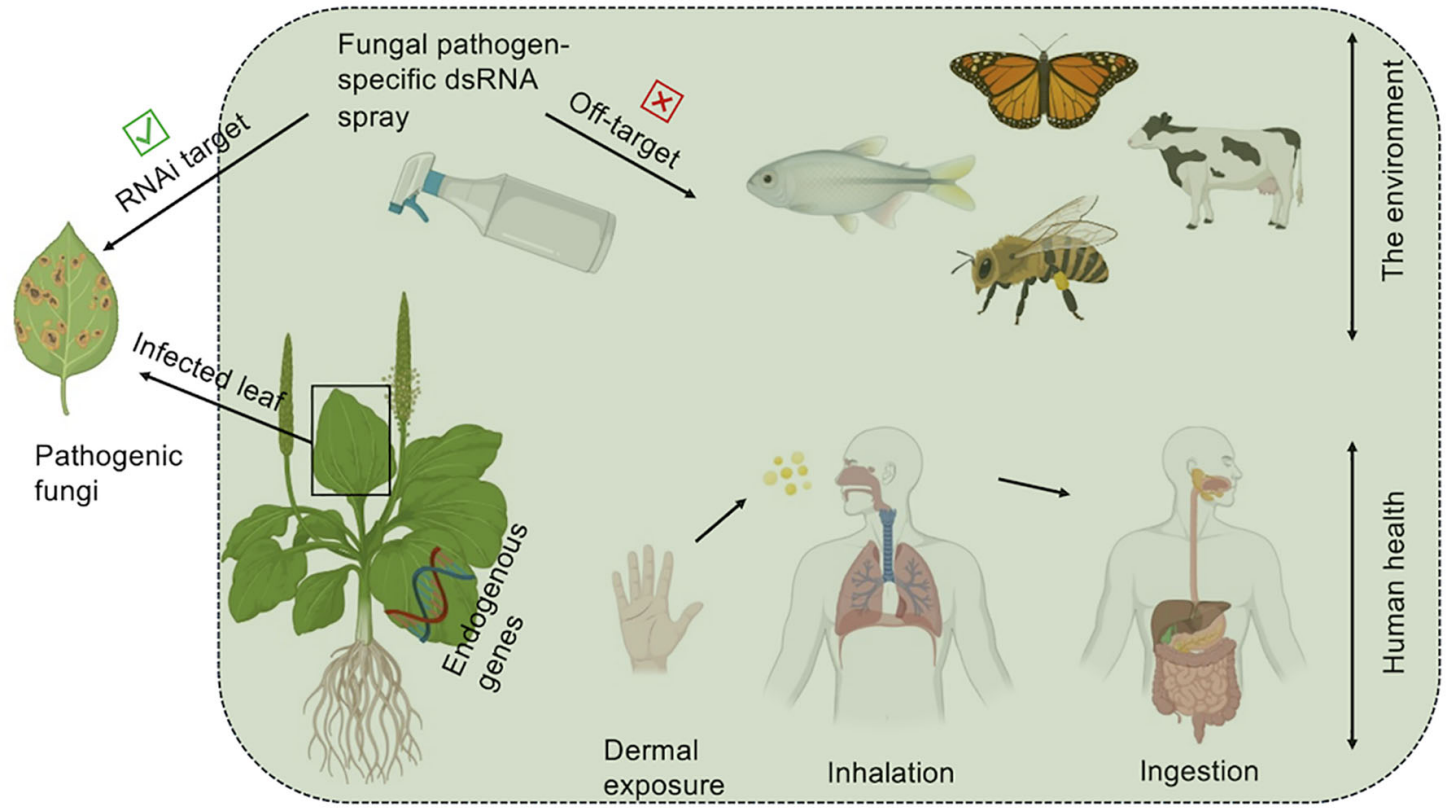
Fungal pathogen-specific dsRNA spray should be effective against specific fungal pathogens while avoiding unintended adverse consequences on off-targets, such as host endogenous genes, and other non-target organisms, including humans (shown inside the green coloured box). Figure adopted from (Fletcher et al., 2020) as per under CC-BY 4.0.

Fungal pathogens are responsible for substantial crop losses globally, affecting key agricultural commodities such as wheat, maize, rice, and fruits. Conventional control methods, including fungicide application and breeding for resistant cultivars, are increasingly undermined by environmental concerns, fungicide resistance, and regulatory challenges. RNAi provides a highly specific and environmentally sustainable alternative by targeting only essential fungal genes, reducing off-target effects on non-pathogenic organisms (**Figure 2**) (Mann et al., 2023).

A notable approach in RNAi-based pathogen control is host-induced gene silencing (HIGS), where plants are genetically engineered to produce dsRNA that targets key fungal genes. Upon infection, the fungus takes up these dsRNAs, initiating RNAi and suppressing pathogenicity. This process has been successfully demonstrated in controlling *Fusarium graminearum* (anamorph of *Gibberella zeae* [Schwein.] Petch.; *Nectriaceae)*, which is the causative agent of Fusarium head blight, by silencing genes such as cytochrome P450s (Koch et al., 2013).

An alternative, non-transgenic approach is spray-induced gene silencing (SIGS), which involves the external application of synthetic dsRNA to plant surfaces. This method avoids genetic modification and enables temporary, environmentally safe gene silencing. SIGS has shown its effectiveness against fungal pathogens such as *B. cinerea* and the ascomycete pathogen *Sclerotinia sclerotiorum*, making it compatible with integrated pest management systems (Smagghe, 2025).

Despite its potential, RNAi-based control faces hurdles, including dsRNA stability under field conditions, uptake efficiency across fungal species, and the robustness of fungal RNAi machinery. Innovations such as nanoparticle carriers and bio-formulated sprays are under investigation to overcome these limitations (Mann et al., 2023). Furthermore, emerging studies demonstrate that targeting virulence-related genes like *MET6* and *MsrA* can impair fungal growth and pathogenicity (Hassouni et al., 1999; Saint-Macary et al., 2015; Molina-Santiago and Vela-Corcía, 2025). Additionally, beneficial biocontrol agents are being engineered to produce RNA molecules that silence fungal genes or which boost their own antagonistic capabilities (Liu et al., 2024).

Implementing RNAi technologies at small and large scales presents both opportunities and challenges that must be evaluated within a One Health framework. At the small-scale level, SIGS offers a non-transgenic and accessible option for growers, yet adoption may be constrained by high dsRNA synthesis costs, rapid environmental degradation of dsRNA by UV radiation and nucleases, and the need for repeated applications (Dalakouras et al., 2020; Tian et al., 2023; Luo et al., 2024). In large-scale agriculture, successful deployment requires cost-effective production systems such as microbial biofactories or chloroplast-based dsRNA expression along with regulatory harmonization and scalable delivery technologies, which remain major bottlenecks (Augustyniak et al., 2018; Dubrovina and Kiselev, 2019). Environmental concerns include dsRNA persistence, impacts on soil and phyllosphere microbiomes, and unintentional off-target gene silencing in non-target fungi or beneficial species, raising One Health considerations regarding ecosystem balance, food safety, and long-term biodiversity impacts (Parker et al., 2019; Dietz-Pfeilstetter et al., 2021). Moreover, off-target effects remain a significant concern, particularly in complex agroecosystems where partial sequence homology may affect beneficial fungi, insects, or symbionts (Roberts et al., 2015; Castellanos et al., 2022). Addressing these barriers will require improved dsRNA design algorithms, comprehensive ecological risk assessments, and environmentally safe delivery systems such as biodegradable nanoparticle formulations that minimize exposure beyond the target organism and help in advancing One Health framework.

#### 3.5.3 Microbiome engineering and microbial consortia

Microbiome engineering and the use of synthetic microbial consortia represent innovative and rapidly advancing strategies in the biological control of fungal plant pathogens. These approaches aim to enhance the resilience of the rhizosphere (i.e., the region of the soil immediately surrounding plant roots), by either transplanting disease-suppressive soil microbiomes or assembling defined communities of beneficial microbes with complementary functions (Berg et al., 2016).

Unlike the application of single microbial strains, synthetic consortia can provide more robust and consistent suppression of soil-borne pathogens, particularly under variable environmental conditions. These engineered communities are designed to exploit synergistic interactions among microbes, such as resource sharing, competition with pathogens, or activation of host defence pathways, thereby reducing the likelihood of pathogenic fungal colonization and disease development.

The strategic design and deployment of these microbial consortia are increasingly guided by advances in meta-omic technologies, including metagenomics, metatranscriptomics, and metabolomics, which provide insights into microbial community structure and function (Zhang et al., 2019). Additional tools, such as machine learning and network modelling, are being employed to predict microbial interactions and identify keystone taxa that are critical for disease suppression (Fitzpatrick et al., 2020; Leggieri et al., 2021). These developments are accelerating the rational design of microbial inoculants that can be tailored to specific crops, soil types, or pathogens. When integrated with sustainable agronomic practices such as crop rotation, organic amendments, and reduced chemical inputs, microbiome-based solutions have the potential to offer long-term and environmentally friendly alternatives to chemical fungicides.

Microbiome engineering raises biosafety concerns related to the unintended spread, persistence, or ecological displacement of introduced microbes or synthetic consortia beyond their target environments. Engineered communities may also disrupt native microbial networks in unpredictable ways, potentially influencing nutrient cycling, non-target organisms, or pathogen evolution. Addressing these risks requires rigorous ecological risk assessment, long-term monitoring frameworks, and regulatory guidelines that reflect community-level and ecosystem-wide dynamics. At the same time, microbiome engineering and synthetic microbial consortia advance a One Health approach by enhancing rhizosphere resilience and reducing reliance on chemical fungicides, thereby lowering risks to human, animal, and environmental health. By harnessing synergistic microbial interactions and tailoring communities to specific crops and soils, these strategies offer sustainable and ecosystem-friendly solutions for durable fungal disease management.

#### 3.5.4 Precision sensing technologies

In parallel with biological developments, artificial intelligence (AI) and precision sensing technologies are improving the timing, placement and efficiency of disease control interventions. AI algorithms using hyper-spectral imaging and remote sensing can detect early disease symptoms and trigger localized treatments with biocontrol agents or RNAi formulations, thereby minimizing resource waste and maximizing protection (Wen et al., 2023).

Despite their promise, these technologies face key limitations. Many remain at the proof-of-concept phase or are restricted to the pilot scale. Challenges include high production costs, complex formulations, regulatory uncertainty (especially for gene silencing and nanomaterials), and variable field efficacy under different agro-climatic conditions. Furthermore, public acceptance and ecological safety, particularly around gene editing and nanotechnology, require transparent risk assessment and regulation.

Key research and policy challenges in advancing novel non-chemical approaches for fungal disease management include the need to better understand the environmental fate and ecotoxicity of nano-materials, particularly their long-term impacts on soil health, non-target organisms, and ecological balance (Schlich et al., 2017; Lead et al., 2018). Also, evaluating off-target effects of RNA interference (RNAi) and genetically engineered microbes is critical to ensure biosafety, especially in complex field environments where unintended interactions may occur (Karaoğlan et al., 2024; Germing et al., 2025). Another pressing challenge is the development of robust and adaptive regulatory frameworks that can keep pace with rapidly evolving biotechnologies, while maintaining public trust and environmental safeguards. Finally, there remains a significant gap between laboratory innovations and practical, field-scale implementation; bridging this requires large-scale demonstration trials, context-specific adaptation, and comprehensive cost-benefit analyses to assess viability and scalability across different agroecosystems. In conclusion, while these advanced tools are not yet replacements for existing practices, they represent a transformative shift toward smart, integrated, and resilient plant disease management systems.

#### 3.5.5 Regulatory, ethical, and biosafety limitations of advanced plant-health technologies through a one health lens

Advanced plant-health technologies such as genome-edited crops, RNAi biopesticides, engineered microbial inoculants, and digital surveillance platforms offer significant promise for sustainable agriculture. However, their integration into agroecosystems highlights persistent regulatory, ethical, and biosafety gaps with implications for One Health, encompassing plant, human, animal, and environmental well-being (**Table 3**).

**Table 3:** Regulatory, ethical, and biosafety limitations of advanced plant-health technologies for fungal disease control through a one health lens.

Technology class	Regulatory limitations	Ethical concerns	Biosafety / ecological risks	One health implications
Genome Editing (CRISPR-edited crops, resistance traits)	Fragmented global regulations; process vs. product-based standards; limited guidance for multiplex editing and epigenome engineering	Public acceptance, transparency, consent in seed systems; equity in access for smallholder farmers	Off-target edits; altered plant–microbe interactions; gene flow to wild relatives	Reduced fungicide uses but potential ecosystem shifts; need for long-term ecological monitoring
RNA Interference (sprayable dsRNA, SIGS)	Lack of harmonized safety assessment frameworks; uncertainty around environmental persistence requirements	Concerns about corporate control, data opacity, and public trust	Unintended effects on non-target fungi or insects; dsRNA stability in soil–water systems	Lower chemical inputs but requires surveillance for non-target gene-silencing effects across environmental compartments
Biocontrol Microorganisms (BCAs, engineered or native)	Slow approval pathways; limited guidelines for synthetic or engineered consortia; inconsistent strain-level regulations	Acceptability of releasing engineered microbes; ownership and benefit-sharing of indigenous strains	Unpredictable establishment; horizontal gene transfer; microbiome disruption	Enhances soil and plant health but requires ecological risk modelling across plant–animal–environment interfaces
Botanical Extracts & Natural Compounds	Variable regulatory classification (pesticide vs. biostimulant); quality-control challenges	Fair access, benefit-sharing, and biopiracy issues surrounding indigenous plants	Batch variability; off-target impacts on beneficial microbes or pollinators	Lower toxicity but inconsistent efficacy may drive overuse or misapplication
Nanotechnology-based Antifungals	Lack of nano-specific agricultural regulations; no agreed metrics for nano-safety	Transparency and risk communication to farming communities	Bioaccumulation; persistence in soil–water systems; toxicity to non-target organisms	Potential to reduce fungicides but may create new environmental health risks
Microbiome Engineering (synthetic communities, targeted microbiome modulation)	Absence of regulatory categories for designed communities; unclear approval requirements for multi-strain products	Manipulating native microbiomes raises questions of ecological stewardship and community consent	Community instability, unexpected metabolic interactions, pathogen evolution	Promotes long-term resilience but requires multi-species ecological impact assessment
Digital & Precision Decision-Support Tools (AI-based disease forecasting)	Data governance, privacy, and interoperability issues; uncertain regulatory oversight for AI-derived decisions	Equity of access; risk of marginalizing low-resource farmers	Over-reliance on AI may reduce local knowledge; model biases	Enhances targeted non-chemical control but risks socio-economic inequity without inclusive design

##### 3.5.5.1 Regulatory gaps and fragmentation

Genome editing illustrates the regulatory inconsistencies that hinder global adoption. Product-based approaches, which exempt non-transgenic edits, contrast sharply with process-based GMO frameworks in other regions, creating uncertainty in trade and biosafety governance (Huang et al., 2016; Wolt et al., 2016; Eriksson, 2018). RNAi biocontrols, although environmentally attractive, confront similar challenges: limited understanding of dsRNA environmental persistence, off-target effects, and trophic transfer complicates risk assessment (Fletcher et al., 2020; Luo et al., 2024; Tardin-Coelho et al., 2025). Engineered microbial consortia amplify these concerns, as traditional single-strain biosafety frameworks inadequately address multi-species interactions, horizontal gene transfer, and unintended ecological consequences (Johns et al., 2016; Duncker et al., 2021; Han and Yoshikuni, 2022). Moreover, digital plant-health systems including AI-driven diagnostics and cloud-linked phenotyping introduce data governance challenges, affecting the monitoring and integration of plant health with broader One Health surveillance (Rose and Chilvers, 2018).

##### 3.5.5.2 Ethical considerations and equity

The One Health perspective underscores the ethical imperative to balance innovation with equity. Proprietary genome-editing tools, RNAi products, and subscription-based digital platforms risk concentrating technological benefits among large agribusinesses, marginalizing smallholders and communities that maintain crop diversity (Huang et al., 2016; Ahmad et al., 2021). Public trust remains fragile when the societal and ecological consequences of these technologies are unclear or poorly communicated. Data governance challenges inherent to digital phenotyping further exacerbate these inequities, potentially redistributing decision-making power away from local stakeholders.

##### 3.5.5.3 Biosafety challenges

Ecological and biosafety risks remain central. RNAi biopesticides exhibit variable persistence and potential off-target effects on beneficial insects, soil microbiota, and aquatic organisms (Petrick et al., 2013; Zotti and Smagghe, 2015; Joga et al., 2016; Fletcher et al., 2020). Genome-edited crops with altered immunity traits may unintentionally influence pathogen evolution or disrupt ecological interactions, cascading across ecosystems (refs. 2,4). Engineered microbial inoculants may disperse beyond intended fields, exchange genes with native microbiota, and alter soil and nutrient cycles, affecting ecosystem functions foundational to One Health (Duncker et al., 2021; Bittleston, 2024). Collectively, these uncertainties highlight the interconnected vulnerabilities **across** plant, animal, and human health systems.

##### 3.5.5.4 Towards responsible one health governance

Bridging these gaps requires harmonized, risk-proportionate governance. Emerging frameworks for genome editing and dsRNA biocontrols provide a starting point for structured risk assessment (Rodrigues and Petrick, 2020; Vengadesen et al., 2025). Ethical deployment depends on transparent communication, inclusive stakeholder engagement, and equitable access. Integrating ecological monitoring, cross-disciplinary collaboration, and open-data infrastructures can align technological innovation with One Health priorities, ensuring that plant-health advances enhance ecosystem, animal, and human resilience while minimizing unintended consequences (Rüegg et al., 2017, 2018).

## 4 Integration into disease management frameworks

**Figure 3:**
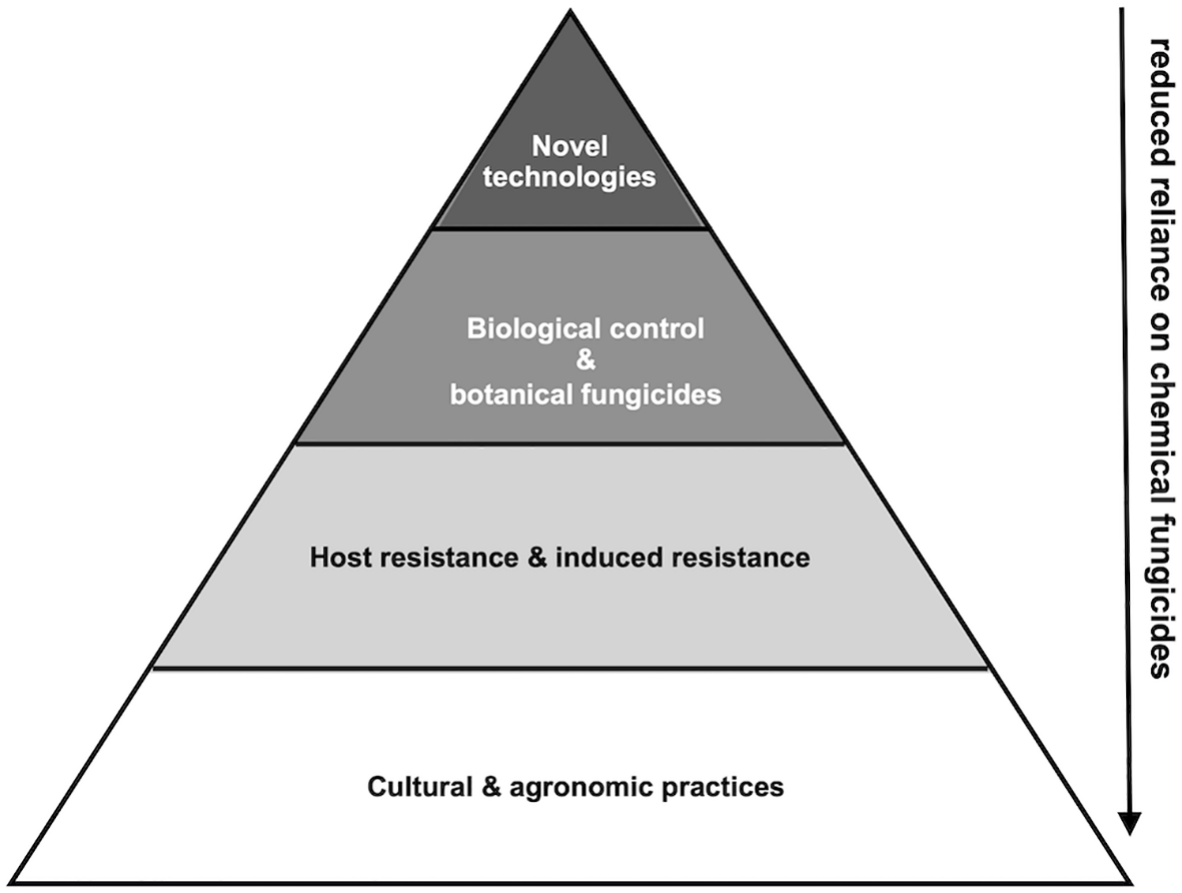
Integration pyramid of non-chemical fungal disease control. A layered framework for deployment of non-chemical approaches to fungal pathogen control, showing foundational agronomic practices, host defence strategies, biological/botanical suppression, and advanced precision tools. This will eventually result in reduced use of chemical fungicides.

No single control method is likely sufficient by itself to manage fungal diseases reliably under complex and high-pressure field conditions. In this scenario, adapting an integrated disease management (IDM) framework is more reliable (Jacobsen, 1997; Deguine et al., 2021). Conceptually, one can envisage non-chemical control as a layered pyramid: at the base, cultural and agronomic practices reduce pathogen inoculum and risk; above these, host resistance and induced resistance bolster plant defences; next come biological controls and botanical fungicides, which act either directly upon the pathogen or indirectly through the plant/microbiome; and at the apex, advanced precision technologies for targeted intervention can be utilised when needed (**Figure 3**). Overlaying all of these layers, continuous monitoring, early-detection, and decision-support systems are managed by combining artificial intelligence (AI) and sensor technologies. In this model, chemical fungicides (when required) should be reserved primarily as a treatment of last resort or as spot treatments, to reduce reliance rather than affect the complete elimination of their use (Deguine et al., 2021; McLaughlin et al., 2023). Synergies and sequencing among these layers can yield substantial benefit. For example, reducing inoculum through sanitation and crop management (cultural layer) can enhance the colonization success of a microbial biocontrol agent; likewise, combining botanical sprays with induced resistance treatments may enhance overall efficacy. The order of deployment matters: as a first step, reducing pathogen pressure through inoculum-lowering approaches: as a second step, applying microbial or botanical treatments prophylactically; and last, utilizing precision monitoring and targeted technologies when thresholds are approached. Integrating weather-based forecasting systems into integrated pest management (IPM) enables early prediction of plant disease risks by combining meteorological data such as temperature, humidity, and rainfall with AI and machine learning models. These systems identify periods of high crop susceptibility, allowing timely and targeted interventions that reduce pathogen establishment and unnecessary chemical use. Recent studies highlight that coupling internet-of-things (IoT) based weather monitoring with AI-driven analytics enhances the precision and sustainability of disease management (Newlands, 2018; Fenu and Malloci, 2021; Delfani et al., 2024). Deployment must also be tailored to different cropping systems, given that organic-production systems may rely almost entirely upon non-chemical strategies, whereas high-input conventional systems may use them to reduce fungicide applications and delay resistance development (van Bruggen et al., 2016; Niu et al., 2020; Deguine et al., 2021). Economic and adoption considerations are critical: lower-risk, easy-to-adopt practices (such as crop rotation) tend to gain faster adoption than more complex microbiome engineering; while ensuring consistent field efficacy, scaling production of microbial or botanical agents, and providing farmer training are essential (Batista and Singh, 2021). Finally, robust monitoring, evaluation and adaptive management underpin the system: tracking pathogen levels, beneficial microbial populations, and environmental variables; using decision-support tools; and employing feedback loops to adjust practices over time (Waylen et al., 2019). Applying One Health criteria to this framework underscores how each management layer contributes to sustainability, risk mitigation, and agroecosystem resilience. Strategies that reduce inoculum or strengthen soil and microbiome function enhance ecological stability, while targeted tools such as induced resistance, biological agents, and precision monitoring minimize risks to human, animal, and environmental health. Framed in this way, integrated disease management offers a coherent path toward crop-protection systems that remain both effective and environmentally sustainable under increasing biotic and climatic pressures.

## 5 Future directions and research priorities

To advance non-chemical control of fungal pathogens into mainstream agriculture, several research and implementation priorities must be addressed. First, large-scale field validation and real-world effectiveness are paramount. While many non-chemical strategies show promise in controlled trials, there is a deficiency of multi-location, multi-year studies that test reliability across various environments, crops and pathosystems (McLaughlin et al., 2023). Understanding context dependency is a critical next step. The performance of biologics, botanicals and microbiome manipulations strongly depends on factors such as soil type, climate, host genotype and cropping history. Research should therefore aim to map this variability and model success and failure cases through meta-analyses and data-driven approaches (Noel et al., 2022). What is equally important are challenges regarding formulation, delivery and shelf life: for microbial BCAs, botanical extracts and nano-based agents, practical issues like stabilizing live microbes, creating shelf-stable products, selecting carriers and designing delivery systems remain major barriers to commercial adoption.

Moreover, microbiome engineering and synthetic consortia represent a frontier in disease management (Wang et al., 2023). By leveraging advances in metagenomics, synthetic biology and systems microbiology, researchers could design tailored microbial communities that suppress pathogens by acting through plant–microbe–pathogen interactions. Yet, this requires a deep systems-level understanding of these complex interactions. In parallel, precision deployment and digital agriculture tools such as early-detection sensors, AI/machine learning, drones- and satellite-based remote sensing and precision delivery systems can enhance timing, targeting and efficacy of non-chemical interventions, which in turn would be supported by real-time decision-making platforms. In addition, the risks of resistance evolution, the implications of climate change and broader sustainability considerations must be integrated into research agenda. Even non-chemical interventions must contend with evolving pathogens and shifting disease pressures under changing climate conditions, emphasizing the need for durability, adaptability and resilience in solutions (McLaughlin et al., 2023).

From a policy and socio-economic perspective, clear and streamlined regulatory frameworks for microbial, biological and plant-derived products are essential. Understanding farmer perceptions, extension requirements, and economic incentives will drive implementation, while comprehensive life-cycle and economic analyses of non-chemical strategies are needed to demonstrate viability. Finally, tackling these challenges demands multidisciplinary and systems-level research combining plant pathology, microbiology, bioinformatics, engineering, agronomy and economics, together with strong trans-disciplinary collaborations between academia, industry and farmers to facilitate adoption and scale-up.

## 6 Conclusion

Non-chemical strategies for controlling fungal plant pathogens are increasingly recognized as essential components of sustainable crop protection systems that align with One Health principles, connecting plant productivity with ecosystem integrity and human and animal health. Cultural and agronomic practices, host resistance, microbial and botanical interventions, and emerging technologies such as RNA interference, nanotechnology, and microbiome engineering provide diverse mechanisms to suppress pathogens while minimizing ecological disruption. Although individually these methods often fall short of the broad-spectrum efficacy of synthetic fungicides, their integration within multi-layered, context-specific management frameworks offers a pathway to resilient, environmentally compatible solutions. Beyond crop protection, these approaches contribute to One Health by reducing chemical residues, preserving beneficial microbiomes, and limiting adverse impacts on non-target organisms, thereby supporting both environmental quality and public health.

Realizing these potential faces multiple hurdles. Technically, proof-of-concept studies must be translated into scalable, consistent field solutions through optimized delivery systems, formulation refinement, and large-scale validation across diverse cropping systems. Integration with precision agriculture, monitoring, and decision-support technologies is critical for adaptive performance. Socio-economic and regulatory challenges including enabling policies, market incentives, farmer engagement, and extension services also determine adoption success. Interdisciplinary research that combines plant pathology, microbiome science, environmental monitoring, and socio-economic frameworks is essential to design interventions that are effective, scalable, and context-sensitive. By addressing these technical and systemic bottlenecks, non-chemical crop protection can progressively reduce reliance on chemical fungicides, enhance ecosystem resilience, and sustain productivity, offering a holistic, One Health-aligned vision for future-ready agriculture.
